# Prediction of the Closed Conformation and Insights into the Mechanism of the Membrane Enzyme LpxR

**DOI:** 10.1016/j.bpj.2018.09.002

**Published:** 2018-09-13

**Authors:** Graham M. Smeddle, Hannah E. Bruce Macdonald, Jonathan W. Essex, Syma Khalid

**Affiliations:** 1 Department of Chemistry, University of Southampton, Highfield, Southampton, United Kingdom

## Abstract

Covalent modification of outer membrane lipids of Gram-negative bacteria can impact the ability of the bacterium to develop resistance to antibiotics as well as modulating the immune response of the host. The enzyme LpxR from *Salmonella typhimurium* is known to deacylate lipopolysaccharide molecules of the outer membrane; however, the mechanism of action is unknown. Here, we employ molecular dynamics and Monte Carlo simulations to study the conformational dynamics and substrate binding of LpxR in representative outer membrane models as well as detergent micelles. We examine the roles of conserved residues and provide an understanding of how LpxR binds its substrate. Our simulations predict that the catalytic H122 must be N*ε*-protonated for a single water molecule to occupy the space between it and the scissile bond, with a free binding energy of −8.5 kcal mol^−1^. Furthermore, simulations of the protein within a micelle enable us to predict the structure of the putative “closed” protein. Our results highlight the need for including dynamics, a representative environment, and the consideration of multiple tautomeric and rotameric states of key residues in mechanistic studies; static structures alone do not tell the full story.

## Introduction

The outer membrane (OM) of Gram-negative bacteria is a formidable barrier to the permeation of molecular species seeking to enter the bacterial cell (1). It is only selectively permeable, enabling molecules essential for the survival of the bacterium such as nutrients to get across the membrane but excluding those that are harmful, such as antibacterial agents. The chemical natures of the lipids of the membrane are thought to play a key role in achieving this selective permeability. The membrane is a lipid bilayer, with the leaflet facing the external environment, known as the outer leaflet, almost exclusively composed of lipopolysaccharide (LPS) molecules, whereas the inner leaflet contains a mixture of zwitterionic and anionic lipids. To date, LPS is perhaps the most chemically complex natural lipid known. The internal membrane component is known as lipid A, which is covalently linked to a polymer of sugars, some of which are phosphorylated. The chemical structure of the LPS molecules varies across bacterial species and sometimes even within one species; for example, in *Escherichia coli* and *Salmonella typhimurium*, the lipid A component has six acyl tails, whereas in *Pseudomonas aeruginosa*, it only has five tails (2). In *E. coli* and *S. typhimurium*, these are laurate and myristate tails in a ratio of 5:1. LPS may be referred to as smooth, rough LPS (Ra LPS), or deep-rough LPS (Re LPS), referring to the number of sugars attached to the lipid A segment. Smooth LPS includes the O antigen and the full core segment, whereas rough LPS contains just the six core sugars and deep rough only two keto-deoxyoctulosonate (Kdo) sugars.

A number of pathogenic bacteria have been shown to synthesize LPS molecules with modified lipid A. Some of these modifications to lipid A can facilitate the development of resistance to drugs; for example, addition of the 4-amino-4-deoxy-L-arabinopyranose group, which is positively charged at pH 7, neutralizes the negative charge of a lipid A phosphate group in *E. coli*, *S. typhimurium*, and *P. aeruginosa*, which reduces the susceptibility of these bacteria to antimicrobial peptides. A number of different bacterial enzymes that catalyze the covalent modification of LPS have been identified. Three of these enzymes, all of which modify lipid A tails, PagP, PagL, and LpxR, are embedded in the OM (3, 4, 5, 6). LpxR catalyzes the removal of two acyl chains of lipid A, in the form of 3-(tetradecanoyloxy)tetradecanoic acid. The X-ray structure of the protein has been resolved to a 1.9-Å resolution, and although the structure of the lipid substrate bound to the protein remains elusive, mutational studies have identified residues that are essential for catalytic activity thus providing clues to the location of the active site (5). The X-ray structure of LpxR was obtained by cocrystallizing the protein with Zn^2+^, and although there is some evidence of Zn^2+^ binding to LpxR in the X-ray structure, the density is low and likely indicative of partial occupation. Interestingly, it has been shown that whereas Ca^2+^ is essential for LpxR activity, some other divalent cations, such as Sr^2+^ and Cd^2+^, but not Zn^2+^ can replace Ca^2+^ without a total loss of catalytic activity (6). Thus, although we know Ca^2+^ is essential, it is unclear from the X-ray structure precisely where it is bound for catalytic activity to occur. Based on their structures and mechanistic predictions, the mechanism of deacylation in LpxR is speculated to be similar to that displayed by a number of phospholipase A2 enzymes (7, 8). The proposed mechanism of deacylation is thought to occur via a histidine, with H122 acting as a base by activating a nearby water molecule, so the latter can react with the substrate lipid molecule to hydrolyze the ester bond. In the X-ray structure, there is a water molecule resolved near H122, which may well be the crucial mechanistic water. Although the structure of LpxR, the location of the water, and the docked lipid substrate provide a plausible static model for the reaction mechanism of the enzyme, the dynamic stability of the model protein-substrate complex, the location of any additional key water and ion binding sites (especially given the very low electron density for Zn^2+^ in the X-ray structure), and the effect of the complex on the local membrane are still unexplored, and thus the mechanism of action has not been proven.

To this end, here we present a molecular simulation study in which we use a combination of molecular dynamics and Monte Carlo simulations to investigate the molecular interactions between LpxR and the Re LPS substrate, with the specific aim of uncovering mechanistic insights into the process of deacylation.

Details of the calculations are presented at the end of the article, but for ease of reading, we present the tables summarizing the molecular dynamics (Table 1) and Monte Carlo (Table 2) simulations below.

**Table 1:** Summary of All Molecular Dynamics Simulations Reported in This Work

System	Notes	Length of Each Simulation	Temperature and Number of Repeats
CG_LpxR	Coarse-grained system	2 *μ*s	310 K (×2)
			323 K (×1)
Apo Model	X-ray structure with water and Ca^2+^ as reported by Rutten et al. (5)	300 ns (×2)	310 K
		1 *μ*s (×1)	323 K
Apo Model^a^	As above but with positional restraints for the first 100 ns	300 ns (×2)	310 K
		1 *μ*s (×1)	323 K
Apo Unbiased	X-ray structure with key water and Ca^2+^ removed from the starting structure	300 ns (×2)	310 K
		1 *μ*s (×1)	323 K
Ligand-Bound Model	X-ray structure with water and Ca^2+^ and modeled in substrate as reported by Rutten et al. (5)	300 ns (×2)	310 K
		1 *μ*s (×1)	323 K
Ligand-Bound Model^a^	As above but with positional restraints for the first 100 ns	300 ns (×2)	310 K
		1 *μ*s (×1)	323 K
Ligand-Bound Unbiased	X-ray structure with modeled in substrate but without water and Ca^2+^	300 ns (×2)	310 K
		1 *μ*s (×1)	323 K
H122_p^b^	H122 is N*ε* protonated	300 ns (×2)	310 K
		1 *μ*s (×1)	323 K
D10A	Single point mutation in protein	300 ns (×2)	310 K
		1 *μ*s (×1)	323 K
D11A	Single point mutation in protein	300 ns (×2)	310 K
		1 *μ*s (×1)	323 K
T34A	Single point mutation in protein	300 ns (×2)	310 K
		1 *μ*s (×1)	323 K
H122A	Single point mutation in protein	300 ns (×2)	310 K
		1 *μ*s (×1)	323 K
Bond Break Model	Scissile bond removed in substrate	500 ns	310 K (×2)
			323 K (×1)
Bond Break Unbiased	As above but with key water and Ca^2+^ removed from the starting structure	500 ns	310 K (×2)
			323 K (×1)
Micelle	Protein in self-assembled DPC micelle	500 ns	323 K (×3)
Apo_DPPC	X-ray structure inserted into simple DPPC membrane	500 ns	310 K (×2)
			323 K (×1)

The total simulation time was 29.6 *μ*s.

a In these systems, the protein, Ca^2+^ ion, and modeled water molecule were subjected to positional restraints for the first 100 ns of simulation; see Methods for more details.

b H122 is N*ε* protonated in this simulation, whereas it is N*δ* protonated in all other simulations.

**Table 2:** Summary of All GCMC Simulations Reported in This Work

Simulation	Repeats	B-Value(s)	Production Steps (Millions)	GCMC Box
H122*ε*	3	−40.79 to −10.79	20	0.2 × 0.2 × 0.2 nm^3^
H122*δ*	3	−40.79 to −10.79	20	0.2 × 0.2 × 0.2 nm^3^
H122*ε*^a^	3	−7.94	40	0.73 × 0.49 × 1.16 nm^3^
H122*δ*^a^	3	−7.94	40	0.73 × 0.49 × 1.16 nm^3^
H122*ε*^b^	3	−40.79 to −10.79	20	0.2 × 0.2 × 0.2 nm^3^
H122*δ*^b^	3	−40.79 to −10.79	20	0.2 × 0.2 × 0.2 nm^3^

a These systems refer to the water location identification simulations.

b In these systems, the imidazole ring of H122 was flipped 180°.

## Methods

### Simulation systems

The coordinates of the LpxR X-ray structure (Protein Data Bank (PDB): 3FID), with Re LPS, Ca^2+^ ion, and a single water molecule modeled as described by Rutten et al. (5), were obtained from Piet Gros. The Ca^2+^ ion is positioned such that it replaces the zinc ion that was resolved in the X-ray structure, given that the former is known to be essential for catalytic activity. These initial coordinates were manipulated to set up a series of simulations that are summarized in Table 1. We truncated the LPS molecule at the Re LPS level, because in this study, we are focused on the lipid A binding site of the protein. This is thought to be located at the lipid headgroup interface; therefore, omission of other sugars and the O antigen is a reasonable approximation; additionally, this allows for longer simulations given the smaller system size compared to the inclusion of the full LPS.

The protein, Ca^2+^ ion, and single water molecule modeled by Rutten et al. (5), were retained in all “Model” simulations. The Re LPS molecule was removed from the putative binding site in “apo” simulations, and the Ca^2+^ ion and single water molecule were removed in “Unbiased” simulations. In “Bond Break” systems, the scissile covalent bond of Re LPS was removed, and the termini were protonated, thereby removing two acyl chains from the molecule. The two resultant molecules were parameterized in a manner consistent with the Groningen molecular simulation (GROMOS) 54a7 force field (9). Both molecules were retained in the simulations. Once the LPS molecule had been converted into two smaller molecules, these systems were then subjected to an additional 500 ns of production simulation. Table 1 summarizes the four mutant proteins also studied, which were constructed by mutating the residues of interest using the PyMOL code (10). In “H122_p” simulations, H122 was N*ε*-protonated, rather than N*δ*-protonated as in all other simulations.

The model membrane used in each simulation was a mix of 90% phosphatidylethanolamine, 5% phosphatidylglycerol, and 5% cardiolipin in the inner leaflet and Ra LPS in the outer leaflet. This is the same membrane composition previously used in the study of OmpA (11). This membrane was created by Piggot et al. (12) to reproduce the most common OM composition of *E. coli*. As in the previous studies, Mg^2+^ ions were used to neutralize the system.

### Coarse-grained simulation protocols

Coarse-grained simulations of the protein in an Ra LPS-phospholipid bilayer were performed to determine the preferred orientation of the protein within the membrane. Coarse-grained simulations used the MARTINI force field, with the LPS parameters of Hsu et al. (13). The Parrinello-Rahman barostat was used for semi-isotropic pressure coupling with a time constant of 1 ps. The velocity rescale thermostat was used, with a time constant of 1 ps. The time step for integrations was 10 fs. Coulombic interactions were cut off at 1.4 nm, and van der Waals forces were reduced to zero between 0.9 and 1.4 nm. Groningen Machine for Chemical Simulations (GROMACS) molecular dynamics software package (version 5.1.4) was used (14, 15).

### Atomistic membrane simulation protocols

Simulations were set up and performed using the GROMACS molecular dynamics software package (version 5.1.4) with the GROMOS 54a7 force field (9, 14, 15). The parameters for Re LPS and Ra LPS are identical to those described by Piggot et al. (12) and Samsudin et al. (11), respectively, and the equilibrated protein-free membranes were taken from these earlier studies. The protein was embedded in the membrane using the GROMACS membed tool (12, 15, 16). The simple point-charge water model was used throughout the simulations (17). Systems were maintained at temperatures of either the biological 310 K or slightly higher at 323 K to improve sampling, using the Nosé-Hoover thermostat with a time constant of 0.5 ps (18, 19). The pressure of the system was maintained at 1 atm, with a time constant of 5 ps, using semi-isotropic pressure coupling with the Parrinello-Rahman barostat (20, 21). The same pressure coupling and barostat were used for NPT equilibration as well as production runs. All van der Waals interactions were cut off at 1.4 nm, and a smooth particle mesh Ewald algorithm was used to treat electrostatic interactions with a short-range cutoff of 1.4 nm. Simulation parameters were chosen based on similar published studies of OmpA (11). Each system was subjected to 500 ps of NVT simulation, followed by 20 ns of NPT for equilibration purposes. Positional restraints (1000 kJ mol^−1^ nm^2^) were placed on the C*α* atoms of the protein and modeled Ca^2+^ ion and water molecules during NVT and NPT equilibration. For some of the simulations, which are highlighted in Table 1, the restraints were kept in place for the first 100 ns of the production runs. Production runs of 300 or 500 ns were then performed for simulations at 310 K or 1 *μ*s for simulations at 323 K. The results were analyzed using GROMACS (14, 15) tools and in-house scripts. Visualization was performed using the visual molecular dynamics software package (22).

### Atomistic micelle simulation protocols

To study protein structure in a more labile environment, LpxR was placed in a box with 100 dodecylphosphocholine (DPC) molecules, Na^+^ ions to neutralize charge, and simple point-charge water. Using a detergent micelle to study protein structure is a well-established method both in silico and in vivo (23, 24, 25). Positional restraints (1000 kJ mol^−1^ nm^2^) were placed on the C*α* atoms of the protein during NPT equilibration, during which a DPC detergent micelle formed around the protein. DPC parameters were downloaded from http://wcm.ucalgary.ca/tieleman/downloads. Equilibration lasted for 50 ns at 350 K to ensure the micelle remained localized around the protein. Production runs of 500 ns were then implemented at 323 K. Principal component and cluster analyses were performed on the resultant trajectories.

### GCMC simulation protocols

Grand canonical Monte Carlo (GCMC) simulations allow for the determination of both the location of water molecules within a defined region and their binding free energies. GCMC involves simulating in the grand canonical ensemble, that is the *μ*VT ensemble, where *μ* is the chemical potential of the system. The chemical potential of the simulation controls the water occupancy of a GCMC region. A *B* value, proportional to the chemical potential will be used from this point forward, because it encapsulates both the chemical potential and additional constant parameters. A detailed explanation of these simulations, including a relation of *B* value and chemical potential have been published by Ross et al. (26, 27). Two types of GCMC simulations were performed; one was performed to calculate the locations of water molecules around the site of esterification using a larger GCMC box, and the other simulations were performed to calculate the binding free energy of the water molecule identified as being likely to be catalytic using a smaller GCMC box.

Within one of the ligand-bound molecular dynamics simulations at 100 ns, H122 was seen to move closer to the scissile bond, from ∼0.9 to 0.6 nm. A snapshot of the system was taken from this and used as the starting point for GCMC simulations using Prototype Molecular Simulation (28). The membrane was discarded, and the protein-ligand complex solvated in a 4.5-nm sphere of the transferrable intermolecular potential four point of water (29). The membrane was discarded to simplify calculations; because the GCMC box was to be placed over an area accessible by bulk solvent, it was unnecessary to include additional lipids. The protein and ligand were modeled using the Amber 14SB and gaff16 force fields respectively (30, 31, 32). A Ca^2+^ ion (parameters taken from (32)) was included in the simulation, where the location of the ion was taken from our atomistic (AT) trajectories. Given H122 is key for the catalytic mechanism, to identify the correct protonation state and provide clues as to its mechanistic role, two sets of simulations were performed (one in which the N*δ* was protonated and the other in which N*ε* was protonated). Because of the similar electron density of carbon and nitrogen, they are difficult to differentiate between in crystallographic electron density. This means that it can be difficult to resolve the orientation of histidine residues experimentally. For this reason, the alternative rotameric states (referred to as the flipped forms) of both the N*δ* and N*ε* will be considered in the binding free-energy simulations. Two equilibration stages were performed; each stage involved one million MC moves. The first equilibration stage involved only GCMC sampling moves, while the second equilibration stage involved both GCMC sampling and sampling of the protein, ligand and bulk water. The protein was sampled as fully flexible with both backbone and side-chain moves. For the larger GCMC box, 40 million production steps were performed, whereas 20 million production steps were performed for the binding free-energy calculations. For the binding free-energy simulations, in which multiple *B* values are simulated, replica exchange in B was attempted every 100,000 steps (27). A list of all GCMC simulations can be found in Table 2.

For location simulations, a large GCMC box of dimensions 0.73 × 0.49 × 1.16 nm^3^ was placed over the region of mechanistic interest, i.e., the H122 and the Re LPS ester group. This simulation was performed to determine the hydration sites over the region. GCMC was performed at the equilibrium *B* value, −7.94, following the Prototype Molecular Simulation default methodology (28). Three independent repeats were performed for both of the H122 tautomeric states.

In addition, for binding free-energy simulations, GCMC calculations were performed to calculate the binding free energy of the specifically identified likely catalytic water molecule. The GCMC titration calculations were performed over a range of chemical potentials (16 equally spaced *B* values over an inclusive range of −40.79 to −10.79) for N*δ*, N*ε*, N*δ*-flipped, and N*ε*- flipped H122 conformations. The water site of catalytic interest was defined as the water position found for the *ε*-protonated H122 of the larger GCMC box simulation. A small cubic GCMC box of length 0.2 nm was used, with the center of the box taken as the centroid of the water molecule. Three repeats were performed, and the binding free energy is calculated by performing 100 bootstrapping calculations of the data.

## Results and Discussion

**Figure 1:**
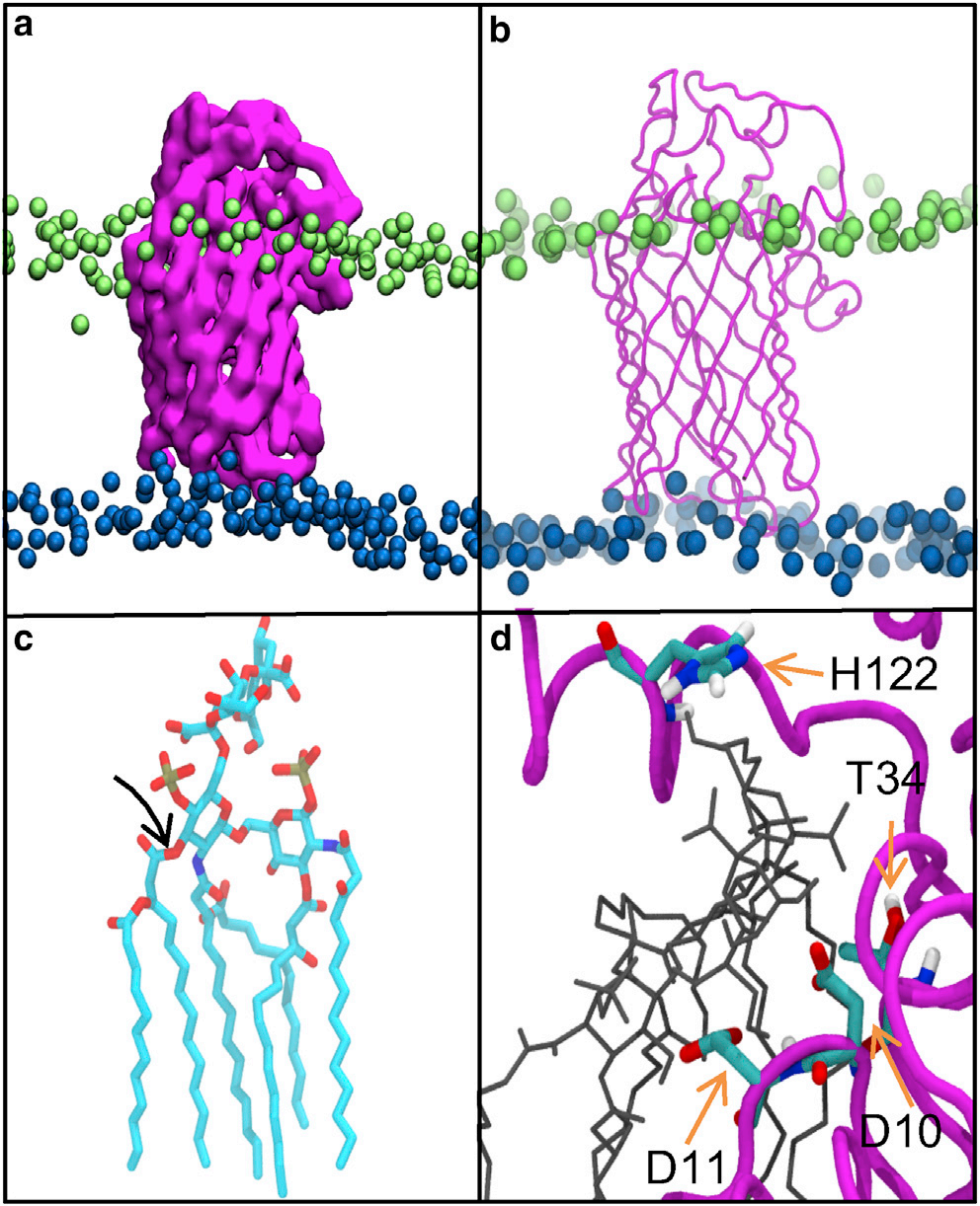
(*a*) Coarse-grained and (*b*) united-atom models of LpxR from the X-ray structure (PDB: 3FID) embedded within a model of the OM. Ra LPS headgroups are shown in lime, phospholipid headgroups are shown in blue, and lipids tails are omitted for clarity. (*c*) Re LPS with scissile bond are indicated and hydrogen atoms are removed for clarity. (*d*) A close-up of the docked Re LPS molecule (*black*) with residues thought to be key for catalysis through mutagenesis studies is highlighted (5). To see this figure in color, go online.

The protein and simulation setup is presented in graphical form in Fig. 1. Positioning of coarse-grain and united-atom LpxR models with respect to phosphate headgroups can be seen in Fig. 1, *a* and *b*. The covalent bond broken when LpxR catalyses the removal of two acyl chains of lipid A is highlighted in Fig. 1 *c*, and a summary of the mutational residues that are key for catalytic activity are shown in active site (Fig. 1 *d*).

### Protein orientation and stability

Coarse-grained simulations were performed to provide an unbiased prediction of the localization and orientation of LpxR with respect to the OM. The densities of molecular components as well as the tilt angle of the protein with respect to the plane of the membrane calculated from the coarse-grained simulations were used to set up the AT systems. Comparisons of these metrics between AT and coarse-grained simulations are provided in the Supporting Materials and Methods. The principal axis of the protein barrel remains tilted by ∼13° relative to the bilayer normal during simulations at both resolutions (this is in close agreement with the tilt angle of ∼16° calculated from the snapshot deposited in the membrane protein simulation database, MemProtMD (33); the differences arise from the latter being simulated in a symmetric phospholipid membrane and thus missing some of the key LPS-protein interactions included in our simulations). As has been reported for other outer membrane proteins (OMPs), the orientation of the protein is maintained in part by the aromatic residues on the outer surface of the barrel being positioned at the lipid headgroup-tail interface of the membrane (34). In the Supporting Materials and Methods, we provide a heatmap of protein interactions with Ra LPS molecules, in which a contact is defined as an interatomic distance of ≤0.4 nm (Fig. S1).

Next, we calculated the area per lipid for both leaflets in our apo-protein systems to ensure well-equilibrated systems. Taking the area per lipid in the inner leaflet of our asymmetric membrane before protein insertion and an average after two independent apo-protein simulations (1-*μ*s simulation time for each) reveals an increase of only 0.01 nm^2^, suggesting little influence from the protein on inner-leaflet packing. For the outer leaflet, we calculated the area per lipid acyl chain, for which the experimental value is 0.26 nm^2^ for LPS tails (35). We calculate an area per LPS acyl chain of 0.28 nm^2^, which is an increase of 0.01 nm^2^ from the membrane before protein insertion and in excellent agreement with the experimentally determined value.

Interestingly we observe some local deformations of the membrane; specifically, the membrane is ∼0.8 nm thinner within a radius of ∼1.2 nm around the protein, compared to further away in what can be considered the bulk lipid region (Fig. S2 *a*). Previous simulation studies of bacterial OMPs have reported thinning of the local membrane, driven by the need to match the width of the hydrophobic region of the membrane with the hydrophobic surface of the protein (36).

A density profile of the molecular components of the simulation systems (Fig. S2 *b*) does not suggest any particular phospholipids are involved in the membrane thinning process. Indeed, comparison of a membrane-only system to our data from apo LpxR in an Ra LPS-containing membrane shows that phosphate headgroups of phosphatidylethanolamine, phosphatidylglycerol, and cardiolipin remain at a distance of 2 nm from the bilayer core despite the presence of the membrane-thinning protein.

Comparison of the order parameters of LPS acyl tails within 0.5 nm of the protein and in the bulk lipid region provides further evidence of the extent of membrane distortion caused by the protein (Fig. S3). There is greater ordering of the acyl tails away from the protein, and those closest to the protein are more disordered. Taken together, these results reveal a significant effect of the protein on the structure of the local membrane. Given LPS has a slow rate of diffusion compared to phospholipids, and often penetration of molecules into the monolayer is rarely observed in MD simulation (37, 38, 39), the structural effects observed here are notable and imply nuanced analysis of these membranes is needed to tease out the effect of local lipids.

Root mean-square deviation (RMSD) and root mean-square fluctuations (RMSF) revealed LpxR to be stable within the OM, both in the apo form and when in complex with Re LPS. The RMSD of the barrel was 0.1–0.15 nm for both bound and apo forms of the protein; these values are similar to those reported from other simulation studies of OMPs (40, 41) (Fig. S4). There was a marked decrease in RMSD of the *α*-helical regions of the protein from 0.3–0.35 nm to ∼0.25 nm when comparing apo to bound forms of the protein. Given the RMSD fluctuates at about the same plateau value at 1 *μ*s as it is at 300 ns, we can be confident that our 300-ns simulations are as valid as the 1-*μ*s simulations for calculating equilibrium properties of the protein.

The extracellular loops showed greater flexibility than the barrel in terms of RMSF, which agrees with other previously reported OMPs (16, 42); this is expected because the extracellular residues are not afforded the same scaffold-like support as the barrel by bulk lipid tails. Details of consistency in tilt angle and system partial density between coarse-grained and united-atom simulations along with further information on RMSD and RMSF can be found in Fig. S4.

### Conformational dynamics of the apo protein

**Figure 2:**
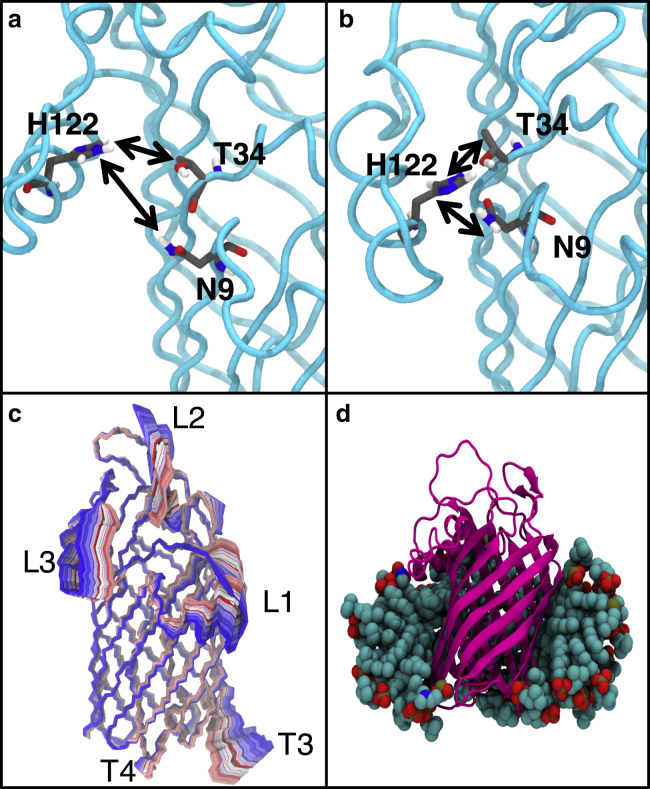
Conformational dynamics of LpxR within a detergent micelle at 323 K. (*a*) The position of catalytic residues in the binding pocket in the energy-minimized LpxR structure (PDB: 3FID). The protein backbone is shown in cyan, with carbon, oxygen, nitrogen, and hydrogen colored gray, red, blue, and white, respectively. (*b*) Closed conformation of the protein revealed by simulations in DPC micelles, with catalytic residues within hydrogen-bonding distance of each other. (*c*) The motion of the protein is depicted by extrapolating between the two extreme projections described by eigenvector 1 and then overlaying the conformation of the protein after every 20 ps. The image is colored on a blue, white, and red scale (*blue* at the start of the simulation, through *white* to *red* at the end of the simulation). (*d*) A representative snapshot of the protein in detergent micelle, some of the detergent molecules have been removed for clarity. To see this figure in color, go online.

Given the slow diffusion rate of LPS (39), to study the conformational states of the protein, we used two alternative environments (a detergent micelle environment and a symmetrical phospholipid bilayer, with the former considered first). Detergent micelles are often used in NMR studies of OMPs and are known to retain the stability of the protein barrels while showing the conformational flexibility of the extracellular loops in the ensemble of structures that is generated (3, 42). Previously reported simulation studies have shown that the labile nature of detergent micelles allows for faster conformational dynamics (38, 41, 43). Over the course of three independent 500-ns simulations at 323 K (labeled “protein in self-assembled DPC micelle” in Table 1), we observed substantial conformational rearrangements of the protein. Whereas the *β*-barrel retained its original conformation as expected, the extracellular loops rearranged such that access to the LPS-binding pocket (Fig. 2, *a* and *b*) was occluded. Furthermore, residues previously identified as key for the catalytic process, N9, T34, and H122, were observed to form intermolecular hydrogen bonds. Thus, a putative closed conformation of the protein has been identified from our simulations.

To further investigate the conformational space sampled by LpxR in the micelle, we performed principal component analysis on the protein backbone by analyzing a concatenated trajectory of all the micelle simulations. The first principal component accounted for 31.5% of the total variance in the backbone. The motion represented by this principal component was movement of extracellular loops L1, L2, and L3, which constitute the walls of the LPS-binding pocket to the “closed” conformation of the protein (Fig. 2 *c*). Furthermore, secondary structure analysis confirmed the stability and conformational integrity of the *β*-barrel segment of LpxR (Fig. S5).

The protein was also simulated within a symmetric DPPC bilayer (three independent simulations of 500 ns duration each) to allow for conformational changes that may lead to the closed state, given these lipids are faster diffusing than LPS. The putative closed conformation identified from the micelle simulations was reproduced in the phospholipid bilayer (Fig. S6 *a*). Interestingly, in simulations of the apo-protein-within DPPC bilayers, L2 retained the *α*-helical conformation observed in the X-ray structure, in contrast to in the micelle simulations. In the DPPC bilayer, very little movement of this loop was observed (Fig. S6 *b*), with residue backbone atoms having an RMSD of 0.34 nm, compared to 0.23 nm in Ra LPS. This is most likely due to the lateral packing of LPS sugar moieties around the extracellular regions of the protein. Principal component analysis was also performed on the protein backbone in DPPC and asymmetric membranes, and two-dimensional projections across the first two eigenvectors can be seen in Fig. S7 *a*. As expected, a smaller area of the conformational space was sampled when the protein was in the more structured environment of the bilayer. That is to say, the DPC micelle enabled the greatest conformational flexibility, followed by the DPPC membrane and finally, the Ra LPS membrane allowed the least. Images of conformational change in the apo-protein backbone in DPPC and Ra LPS membrane are provided in Fig. S7, *b* and *c*.

### Cation binding sites in the apo protein

Having established the structural stability of the protein, we then characterized the behavior of the apo protein with respect to the putative Re LPS and cation-binding sites. We looked for evidence of a hydrogen bond between the carboxylate side chain of E128 and N*ε* of H122, as is suggested in the proposed mechanism. However, across the 4.8-*μ*s simulations of the apo protein in bilayer molecular dynamics simulation, there was little evidence of a stable hydrogen bond here; because of steric hindrance produced by the backbone of extracellular loop L3 (residues 110–139, with the *α*-helix within this loop defined by residues 110–126), it was easier for a hydrogen bond to form between the backbone carbonyl of E128 and N*ε* of H122. The distance between the center of mass of H122 and E128 over 300 ns is shown in Fig. S9 *a*. We return to this later when discussing the mechanism of deacylation.

In each of the “model” simulations, the Ca^2+^ ion moved away from its initial binding site as soon as positional restraints were removed, indicating that either 1) a ligand is required to stabilize the ion within the binding site or 2) this is not the cation binding site. When subject to positional restraints, the Ca^2+^ remained ∼0.25 nm from T34; when restraints were removed, the ion moved away to ∼1.2 nm from T34. The positioning of Ca^2+^ in the model corresponds to electron density attributed to a Zn^2+^ ion in the X-ray structure (PDB: 3FID) (5).

### Cation binding sites in the ligand-bound complex

**Figure 3:**
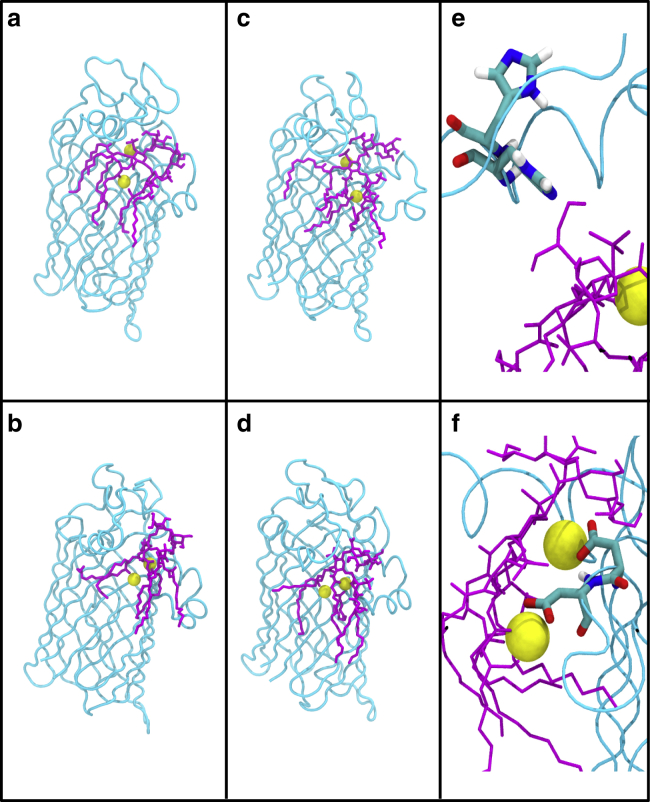
Snapshots of the protein-lipid-ion complex after 300 ns from two independent simulations of the ion-biased (*a* and *c*) and ion-unbiased (*b* and *d*) simulations. The two rotameric states of H122 are shown in (*e*), and (*f*) shows coordination of cations by residues D10 and D11. The protein is colored cyan, Re LPS is magenta, and ions are yellow. The membrane, water, and other ions are omitted for clarity. To see this figure in color, go online.

The protein-ligand membrane system was simulated in several states (Table 1); each one was initiated with the protein-lipid complex as predicted by docking calculations reported by Rutten et al. (5). In “Model” simulations, the Ca^2+^ and water molecule proposed to be essential for the catalytic mechanism were retained, whereas in some simulations these were removed. We consider the former first. The Re LPS substrate remained noncovalently bound to the protein through electrostatic interactions between the Kdo-sugar-hydroxyl groups and the basic residues K67 and R68 for the duration of all of our simulations. Interestingly, Reynolds et al. (6) noted that lipid A, which lacks Kdo sugars, is a poor substrate for LpxR. Our results suggest that this is likely due to the absence of the salt bridges between the Kdo sugars and the protein. Positioning of Re LPS with respect to the binding site on LpxR for “Model” simulations may be seen in Fig. 3, *a* and *c* and for “Unbiased” simulations in Fig. 3, *b* and *d*; each image in Fig. 3 was produced from a representative simulation at 323 K.

H122 sampled two major rotamers throughout the simulations (Fig. 3 *e*). The distance between the center of mass of H122 and the scissile bond varied between 0.3 and 0.9 nm, although for most simulations the imidazole of H122 remained in a conformation angled toward the scissile ester bond. As expected, the H122A mutation did not affect protein interaction with Re LPS, nor did it affect the conformation of the L3 *α*-helix (residues 110–126), as shown in Fig. S8 *b*.

Interestingly simulations of the wild-type protein predict a second cation-binding site in which an Mg^2+^ ion is coordinated by the glycerol oxygen adjacent to the scissile bond, the carboxylic acid moiety of D11, and water molecules found persistently in this region throughout the simulations. Coordination of cations by D10 and D11 is shown in Fig. 3 *f*. When the Ca^2+^ ion is removed before equilibration, the conformational behavior of the putative active site is rather different. Although two Mg^2+^ ions are observed to enter the active site region during equilibration and remain within this region throughout the simulations, neither one is located precisely in the same spot as the Ca^2+^ ion from the Rutten model (5). Furthermore, H122 is observed to flip out of the active site such that the side chain is pointing toward the extracellular loops. This movement of H122 is accompanied by snorkeling of K67 toward the phosphate group of the lipid A sugar (Fig. S9) rather than interacting with the hydroxyl groups of the Kdo sugars as observed with the simulations of the Rutten model when Ca^2+^ is located within the proposed binding site (5). Residues T34 and D10 are not involved in interactions with the two cations in these simulations; instead one Mg^2+^ ion is observed to interact with D11 and two glycerol oxygens from adjacent acyl tails. The second cation-binding site identified in the wild-type simulations is again observed here with D11 involved in coordination along with a glycerol oxygen from one of the substrate lipid tails. The D11A mutation leads to the cation moving out of this binding site, indicating the importance of residue D11 for coordination of the second ion. In Fig. S10, we provide heat maps showing protein contacts with the Ca^2+^ ion and Mg^2+^ ions, where contact is defined as an interatomic distance of ≤0.4 nm.

**Figure 4:**
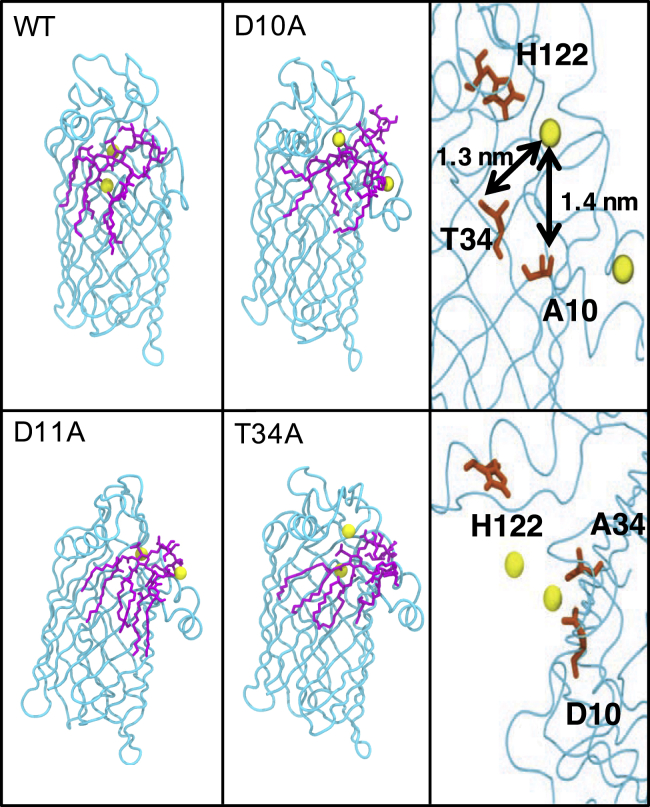
Snapshots of the protein-lipid-ion complex with specific residue mutations after 300 ns, compared to the wild-type. The membrane and solvent have been omitted for clarity. The top right panel shows the cation moving away from residue 10 when it is mutated from D to A. The bottom right panel shows the cation is still near its original location when residue 34 is mutated from T to A. The color scheme is the same as Fig. 3. To see this figure in color, go online.

The scissile bond is oriented through interaction of the carbonyl oxygen with the Ca^2+^ ion; the latter is also coordinated by N9, D10, T34, and three water molecules throughout the simulations of the wild-type protein. In simulations of the protein with the single-point mutation D10A, the Ca^2+^ moved out of the binding site such that after 300 ns it was no longer interacting with the protein, providing strong evidence that residue D10 plays a key role in coordination of the Ca^2+^ ion. In simulations of the T34A mutant however, the ion Ca^2+^ remains in the pocket, still coordinated by D10. Thus, our simulations suggest that for Ca^2+^ ion coordination, residue D10 is essential but T34 is not. Fig. 4 shows the effect of specific residue mutation on the conformation of the protein-ion-lipid complex. Again, all images in Fig. 4 were produced from simulations at 323 K.

Our simulations therefore predict that both the aspartate residues within the putative binding site, D10 and D11, play a role in binding cations. Interestingly, when Ca^2+^ is not coordinated by D10, the protein remains stable and the protein-substrate complex does not dissociate over the timeframe of our simulations; however, the local conformational rearrangements of the nearby residues are such that it is difficult to envisage a deacylation process occurring. As such, our simulations support the hypothesis of Rutten et al. (5) in which D10 must coordinate a cation. However, this does leave open the question of why does D11 also bind a cation? Cation binding in this region is observed in all our simulations (other than those of the D11A mutant); therefore, cation binding by D11 is likely to be important for ion recruitment to the active site.

### Catalytic mechanism

**Figure 5:**
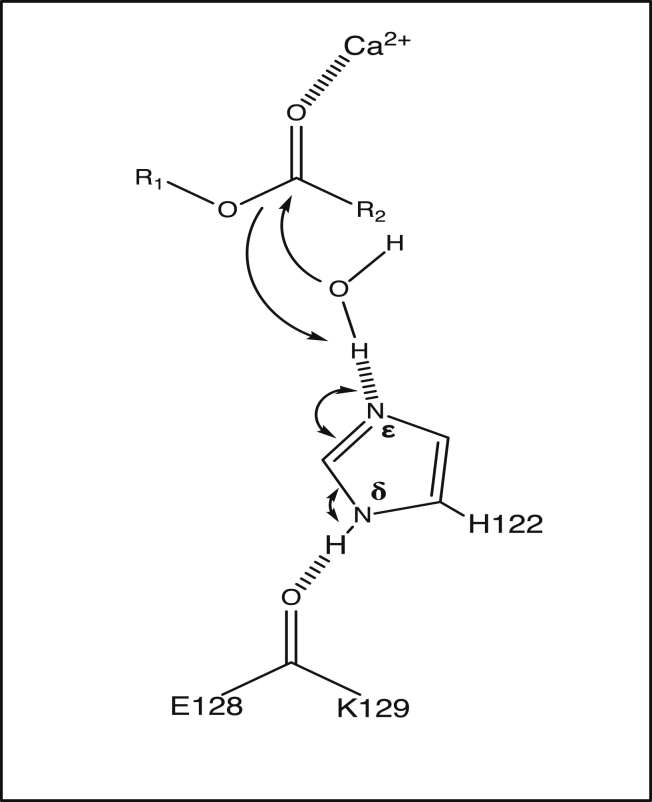
Catalytic mechanism including the residues E128, K129, H122, the portion of Re LPS to be cleaved, and one Ca^2+^ ion.

Having identified the conditions under which cations bind to the enzyme, we next sought to gain some insights into the catalytic mechanism of deacylation by LpxR by combining the results of molecular dynamics and Monte Carlo simulations, with clues from what is known about the mechanisms of phospholipase A2 enzymes (8) and a previously hypothesized mechanism for deacylation from the static X-ray structure, docking studies, and mutagenesis studies (5, 6). It is important to note here that initial predictions do not specify whether the mechanism requires one mechanistic water or a cascade of two water molecules; the latter is known to be the case for the mechanism of a secreted pancreatic phospholipase A2 enzyme (7, 8). Attention was paid to the number of water molecules found between H122 and the scissile ester during GCMC calculations. The putative catalytic protein residues, Ca^2+^ ion, known to be essential for activity, and the ester moiety of the lipid substrate are shown in Fig. 5.

**Figure 6:**
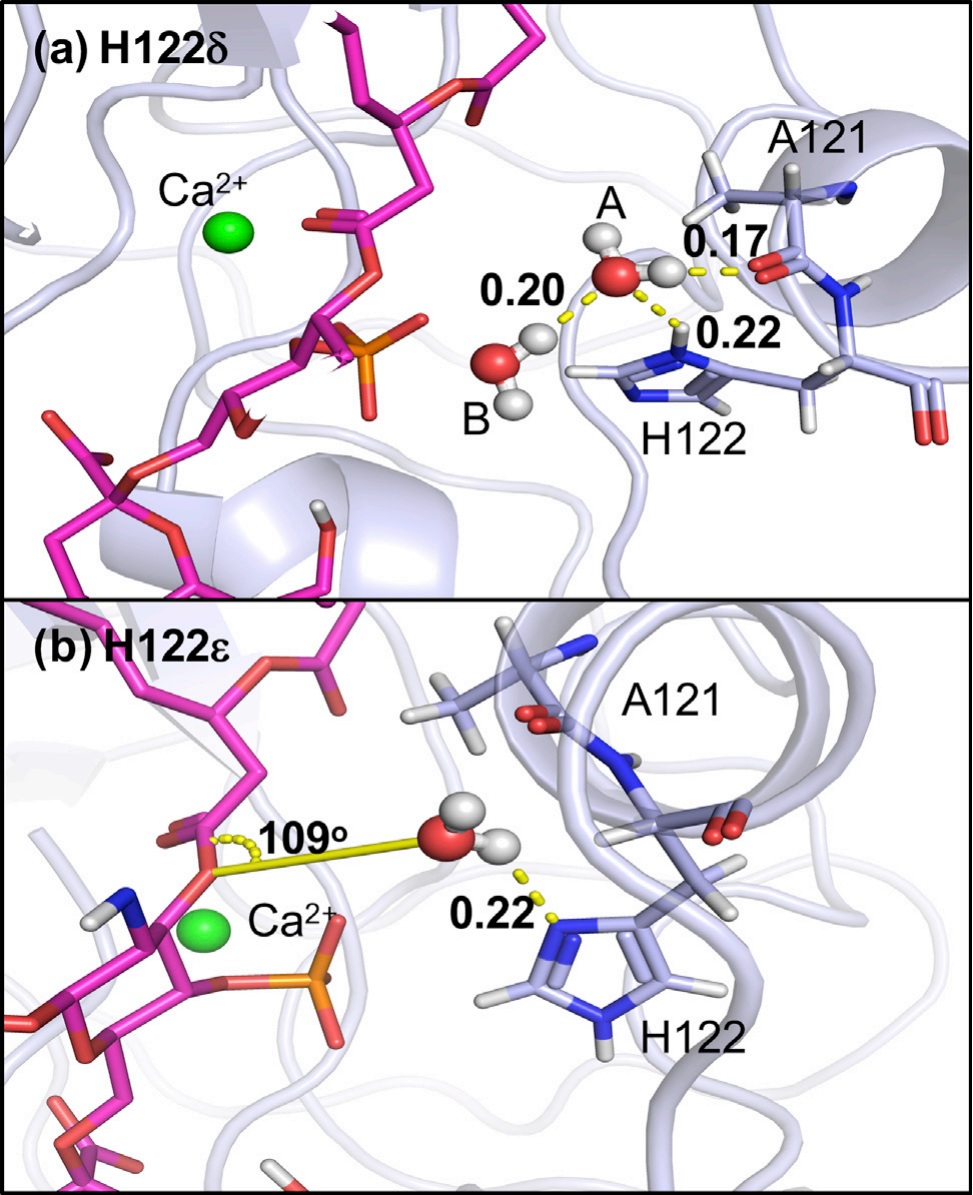
(*a*) Water locations from the GCMC simulation where H122 is N*δ* protonated. Two water molecules found between the H122 and the ester. Hydrogen-bonding interactions are illustrated with a dashed yellow line, with length shown in nanometers. Water molecule B is forming hydrogen bonds with the H122 H*δ*, water A, and A121. The water A molecule is ∼0.3 nm from the ester group throughout the simulation and is orientated with the oxygen atom pointed toward the group, which means that it is unable to hydrogen bond to the ester group and would therefore not result in hydrolysis. The water is stabilized in this orientation by hydrogen bonding to another water close by. (*b*) A snapshot of the N*ε*-protonated H122. A121, H122, and ligand are shown in stick representation, and the protein backbone is shown in light blue. The calcium ion is shown in green. The relevant water(s) in each case are shown in red and white. Part of the ligand and other GCMC water molecules have been removed from the image for clarity. To see this figure in color, go online.

To study aspects of the mechanism of deacylation and to predict the number of water molecules likely to be involved in the mechanism, we performed GCMC simulations to determine the positions of water molecules near the protein-ligand complex. Simulations were performed at *B*_eq_, which corresponds to equilibrium with bulk water. The two protonation states of H122 were once again studied. H122 was more mobile in simulations in which it was N*δ* protonated. In two of the three independent simulations, the H122 shifted its position by rotating away from the Re LPS ester group, leaving a predominantly dry vacancy between H122 and the ligand. In the one independent repeat simulation in which the directional interaction between the H122 and the ester group was maintained, two water molecules were located by GCMC in between the two moieties as part of a chain of water molecules. Although the molecules were suitably positioned, the orientation of the water was such that formation of a hydrogen bond to the ester group of the substrate was not possible. However, the water was able to form a compensating hydrogen bond with another nearby water molecule. A snapshot of this water network and hydrogen-bonding interactions is shown in Fig. 6.

**Figure 7:**
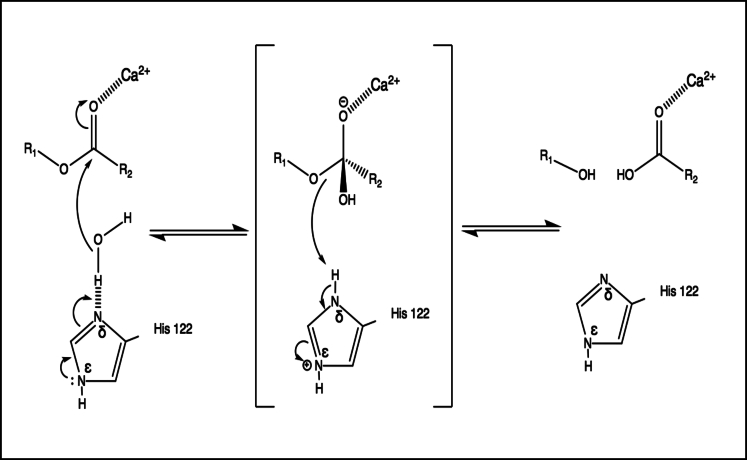
The predicted mechanism of deacylation based on our molecular dynamics and GCMC simulations. The catalytic histidine, H122, is N*ε* protonated. To establish whether the GCMC and molecular dynamics simulations were identifying similar water sites, we defined a 1-nm^3^ box around H122 and the ester moiety of the ligand in the ligand-bound unbiased trajectory. Using visual molecular dynamics, we found that water molecules occupied this space for 95% of our 1-*μ*s molecular dynamics simulation at 323 K. More than one water molecule was found when the distance between H122 and the ester moiety increased.

When the H122 was N*ε*-protonated, the hydrogen-bonding interaction was flipped, with a hydrogen bond accepting N*δ* oriented toward the ester bond. In all three repeats, the GCMC method located a single water molecule interacting with both the H122 and the ester bond. The orientation of this water molecule, shown (Fig. 6) was such that it would enable it to act as a nucleophile in the catalysis. This water molecule was ∼0.2 nm away from His122 (N*δ* to water O) and ∼0.3 nm away from the ester (water O to carbonyl C) throughout the simulation. The oxygen atom of the water molecule was at an angle of 109° to the plane of the ester group, close to the Bürgi-Dunitz angle of 107° (44). Based on the clustering of the GCMC-inserted water molecules, the water was present in this position 82.9% of the time when H122 was N*ε*-protonated. Analysis of our MD simulation over 1 *μ*s revealed that water was present in this region for ∼95% of the simulation, therefore clearly showing the two methods converging to this water site. The binding free energy of the catalytic water was determined using GCMC titration simulations for all combinations of rotameric and tautomeric states of H122, conformations of which are shown in Fig. S11. The water molecule in the catalytic position was found to have binding free energies of N*ε* −8.5 (0.2), N*δ* −5.9 (0.3), N*ε* flipped −6.0 (0.2), and N*δ* flipped −7.0 (0.2) kcal mol^−1^ respectively, with SD shown. The corresponding GCMC titration curves are provided in Fig. S12. In all cases, the water molecule is tightly bound, but the binding free energy is most favorable for the N*ε* conformation (the proposed catalytic conformation). The orientation of this water molecule relative to H122 and the ester bond, as well as its energetic stability, provide compelling support for it being the catalytic water molecule. We note here that Bahnson (8) suggests that the catalytic mechanism of phospholipase A2 involves water activation by a histidine that is N*ε* protonated. Based on the GCMC results favoring the N*ε* protonation of H122 and the results from our molecular dynamics simulations, we can predict a mechanism for deacylation. We hypothesize here that the role of Ca^2+^ in the deacylation is to further polarize the sn2 carbonyl oxygen of the ester bond. The mechanism shown in Fig. 7 has H122 directly increasing the nucleophilicity of the catalytic water via hydrogen bonding from N*δ*. As previously mentioned, although E128 has been hypothesized to stabilize the orientation of H122 through hydrogen bonding, we do not observe a persistent E128-H122 hydrogen bond in any of our simulations, regardless of the protonation state of H122. The lack of hydrogen bonding between these two residues could in fact account for the relatively low activity of LpxR in *S. typhimurium* (6, 45); it remains to be seen whether a modified membrane composition could alter LpxR orientation and conformation, thereby stabilizing the proposed E128-H122 hydrogen bond to increase the basicity of H122 therefore activating the catalytic residue. Further detail of the rotamers sampled in GCMC simulation can be seen in Fig. S13.

### Protein and substrate after deacylation

**Figure 8:**
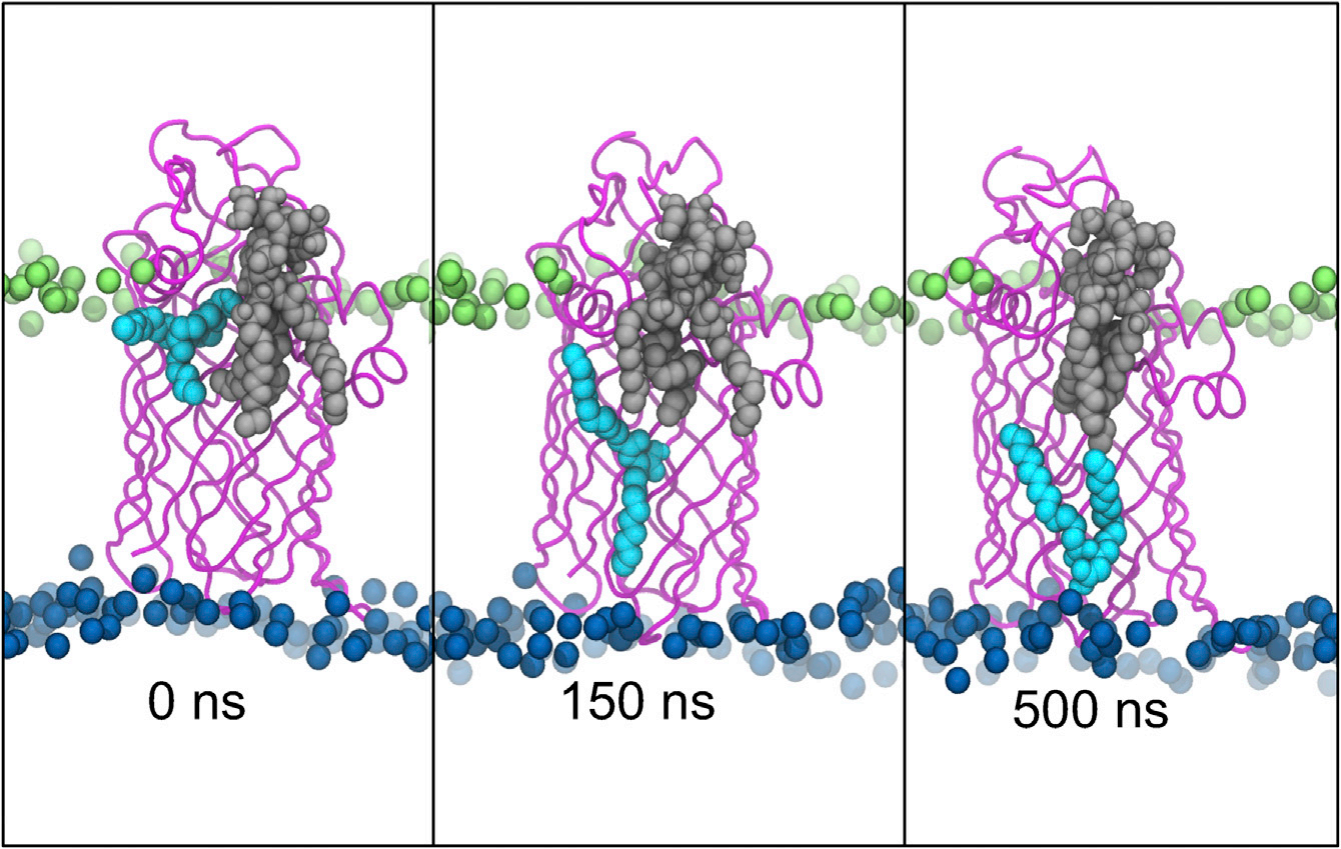
Movement of catalytically cleaved acyl tails (*cyan*) away from the protein (*magenta*) and the truncated Re LPS substrate (*gray*). To see this figure in color, go online.

To predict the diffusion of the products that occurs postcatalysis, the Re LPS ligand was manually deacylated by removing the covalent bond that would be enzymatically cleaved by LpxR. Simulations initiated with the two postcatalysis molecules placed within the hypothesized LPS binding region reveal that the cleaved acyl tails rapidly move away from the protein, toward the lower leaflet of the OM. Meanwhile, the modified LPS molecule remains within the putative binding site for the duration of our simulations. Each one of our six postcatalysis systems show the acyl tails moving toward the inner leaflet within 500 ns, as shown in Fig. 8. Images in Fig. 8 are produced from the bond-break unbiased simulation at 323 K.

In the case of OmpLA, the movement of lipid tails toward the inner leaflet leads to increased permeability and fluidity of the inner leaflet. This plays a role in bacteriocin release; it remains unclear as to whether LpxR could be implicated in the same way or whether the acyl chains are promptly repurposed in lipid synthesis.

It is useful to consider the main limitation of the current study. This arises from the initial enzyme substrate originating from a docked model. Although the simulations show that lipid A is stable in the docked conformation within the putative binding site, structural data would confirm the structure of the enzyme-substrate complex. It is also worth noting that our study does not include polarization and other quantum effects, which would allow for a more detailed study of the chemical reaction mechanism. Having said that, quantum mechanics calculations of membrane-embedded proteins are immensely challenging, with very little available in the literature in terms of best practice. The approach we have used here of studying multiple protonation states of the catalytically active residue and using GCMC to identify water-binding sites provides a sound classical alternative to using quantum methods such as transition-state searching. However, it is clear that such mechanistic studies would benefit from representation at the quantum level, and we are moving toward developing protocols to enable this in the future. We note here that representation of ions with a classical molecular mechanics force field is notoriously difficult given we rely on Lennard-Jones parameters and a fixed charge. Here, we have employed the GROMOS 54a7 force field, in which the Lennard-Jones parameters for Ca^2+^ are unchanged from those in the 53A6 force field. These parameters have been successfully used in a number of studies in which specific ion binding plays a key role in the system dynamics (9, 46, 47). Overall, the models and methods used here come with their caveats, as do all scientific methods, but by using a combination of modeling, molecular dynamics, GCMC, and performing multiple simulations of each system we have ensured that the limitations are addressed as far as possible.

The mechanism of deacylation by LpxR is of immense interest from an immunological and microbiological viewpoint, given that modification of LPS can lead to antimicrobial resistance. Here, we have employed molecular dynamics and Monte Carlo simulations to study the conformational behavior of the protein, which has enabled us to predict the structure of the putative “closed” state of the protein in the absence of a substrate. The closed state of the protein exhibits hydrogen bonds between residues known to be key for the catalytic process. Thus, we hypothesize that in the absence of substrate, these residues play a role in stabilizing the protein in the closed conformation. For the substrate-bound state of the protein, we identify key protein-substrate interactions that hold the substrate within the active site. For the catalysis itself, we show that residue D10 plays a key role in coordinating a divalent cation. Our simulations predict the catalytic histidine to be N*ε* protonated, which differs from the previously proposed mechanism from structural data and docking studies in which the histidine would have to be N*δ* protonated. Furthermore, we provide quantitative evidence that one water molecule occupies the space between the catalytic histidine and the scissile bond in a tightly bound conformation and thus seems suitably placed to participate in the hydrolysis reaction. We show that the tails that are removed from the LPS molecule are able to rapidly diffuse toward the inner leaflet and presumably insert into this leaflet, whereas movement of the remainder of the LPS molecule out of the active site is slower. The combined data from the different types of simulations enable us to hypothesize the full mechanism of catalysis and also the movement of the newly formed chemical species, postcatalysis. Indeed we show the importance of considering the conformational dynamics of membrane enzymes and ligands in a suitable local environment when attempting to decipher their mechanism of action.

## Author Contributions

G.M.S. and H.E.B.M. performed the simulations. G.M.S., H.E.B.M., J.W.E., and S.K. analyzed the data. G.M.S., H.E.B.M., and S.K. wrote the manuscript.

## Supporting Material

Document S1. Figs. S1–S13

Document S2. Article plus Supporting Material
