# Supplementary material for: Prediction of the Closed Conformation and Insights into the Mechanism of the Membrane Enzyme LpxR
Source: Biophys J. 2018 Sep 13;115(8):1445–56. doi: 10.1016/j.bpj.2018.09.002 (PMC6260217; doi:10.1016/j.bpj.2018.09.002)
Supplement: Document S1. Figs. S1–S13 [file mmc1.pdf]

Biophysical Journal, Volume *115*

Supplemental Information

Prediction of the Closed Conformation and Insights into the Mechanism of the Membrane Enzyme LpxR

Graham M. Smeddle, Hannah E. Bruce Macdonald, Jonathan W. Essex, and Syma Khalid

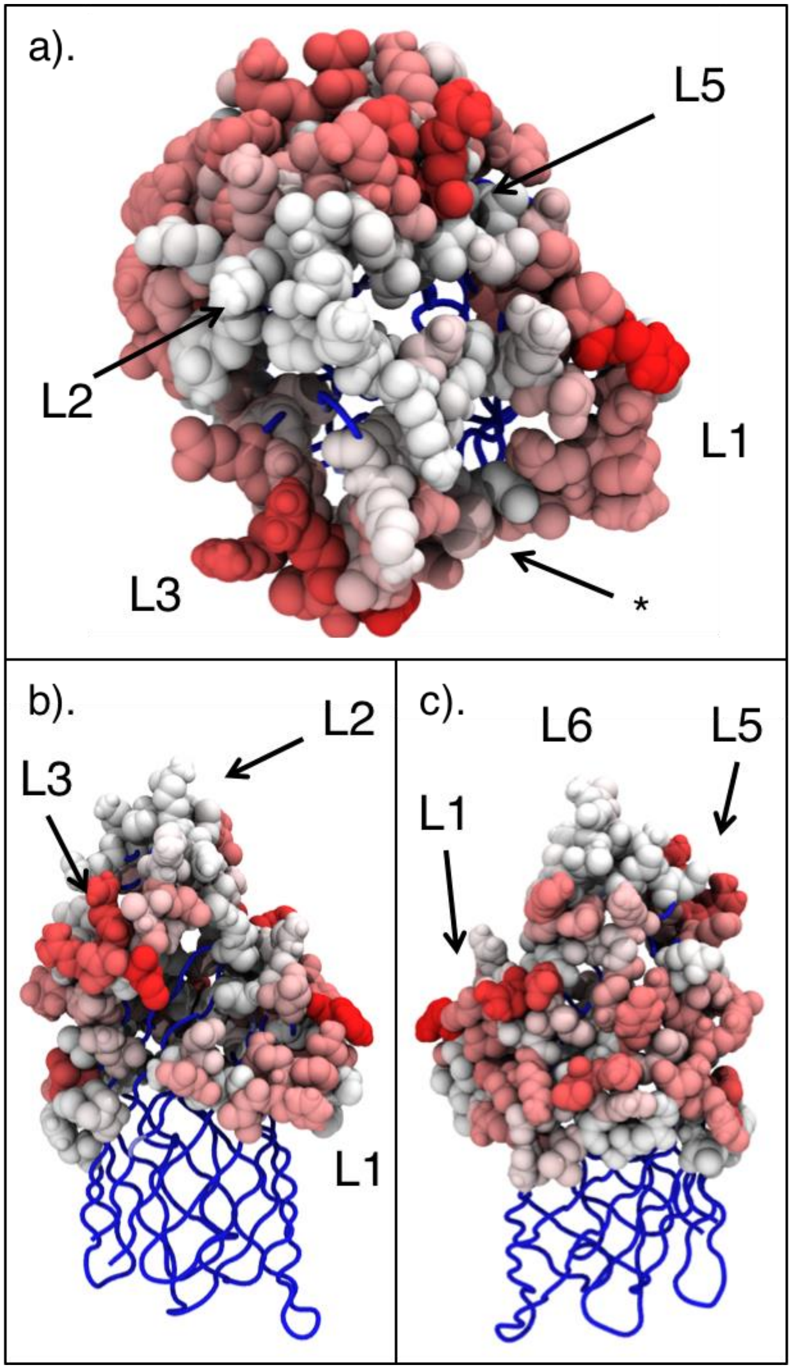

Figure S1. a) Heat-map of protein residue contacts with Ra LPS molecules, with BWR colour scale and extracellular loops annotated. Contact defined as an interatomic distance of ≤ 0.4 nm, and images generated from a representative 1μs *apo* protein trajectory at 323 K. **a)** A top-down view of the protein, with an asterisk denoting the LPS binding site. **b)** viewing LpxR from the side, with direct

focus on the LPS binding site. **c)** viewing LpxR from the opposite side to **b)**, showing area typically immersed in LPS.

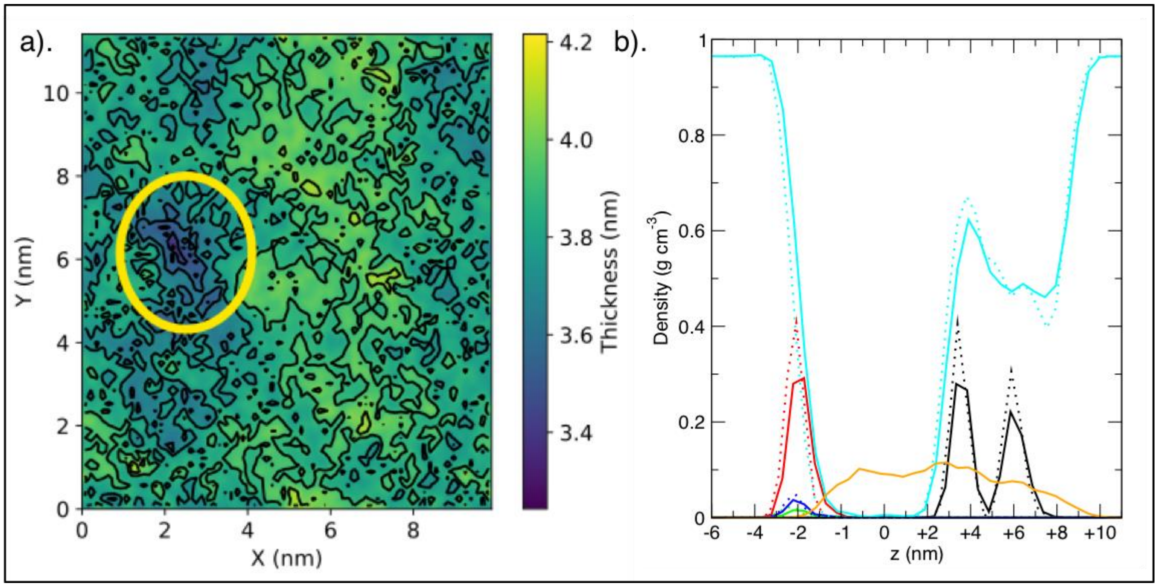

Figure S2. a) Depicting the thickness of the bilayer as a 2D landscape for the membrane. LpxR is shown to decrease the distance between phosphate residues of opposing leaflets in close proximity to the protein. Yellow circle denotes position of LpxR averaged across the simulation. b). Density of Re LPS (black), PE (red), PG (green) and DCG (blue) phosphates, as well as water (cyan) and protein (orange) across the z axis, comparing between membrane only and protein in Ra LPS membrane systems. Solid lines denote protein in membrane system and dashed line membrane only, with vertical black dashed lines highlighting the transmembrane (TM) region

To assess any local membrane deformations caused by LpxR, membrane thickness analysis was carried out on the 2 μs CG_LpxR system at 323 K. Membrane thickness is here denoted as the distance between phosphate positions of opposing bilayers.

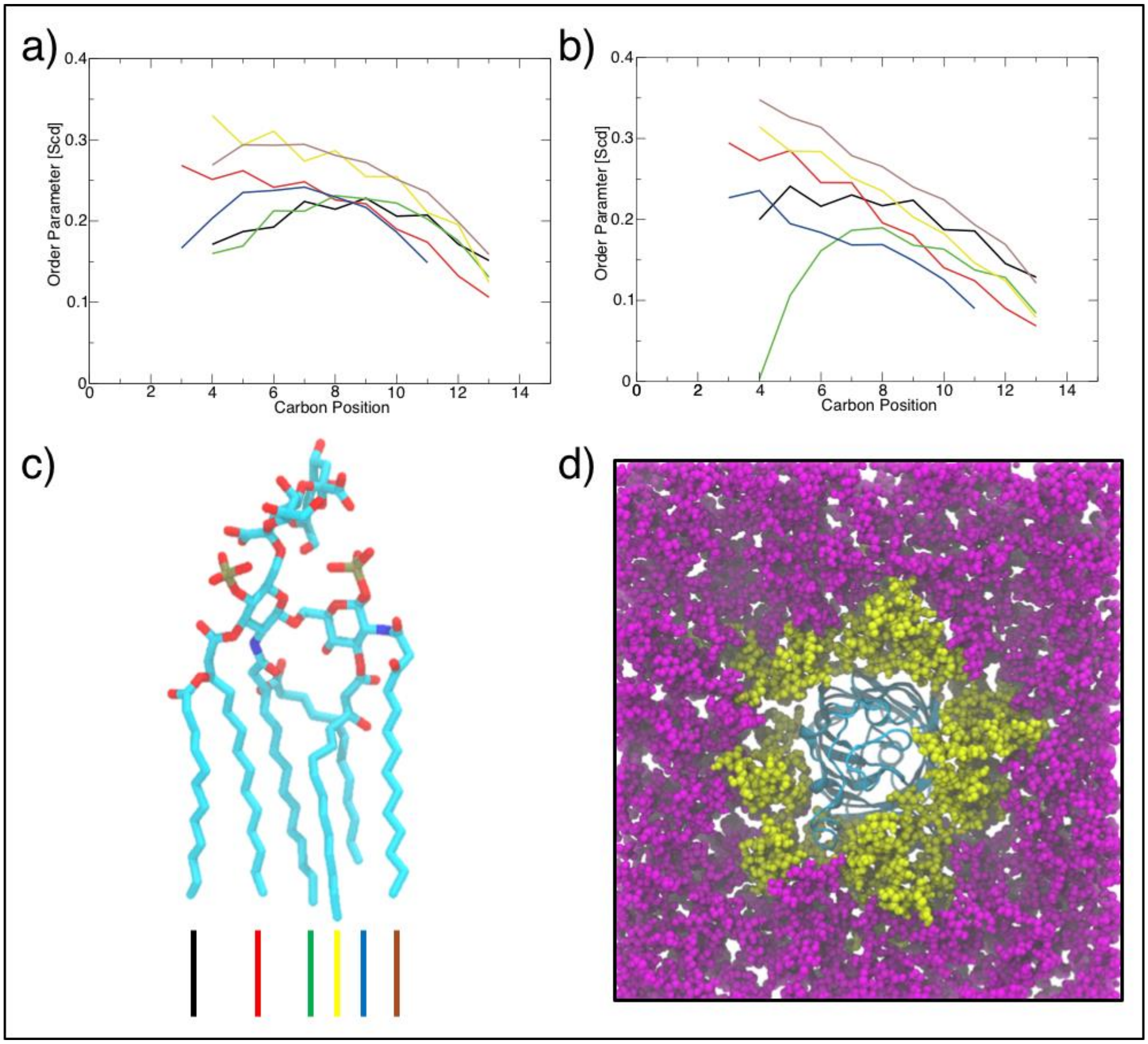

Figure S3. a). Deuterium order parameters of bulk LPS acyl tails during 1 μs *apo* simulation at 323 K. **b).** Deuterium order parameters of acyl tails of LPS within 0.5 nm of LpxR. **c).** LPS molecule with tails colour coordinated to correspond with order parameter plots. **d).** Highlighting LPS residues selected for deuterium order parameter calculation. LPS included in **S2a** in magenta, **S2b** in yellow and protein in cyan.

Figures S3a and **S3b** show that lipid tails are more disordered in the region directly surrounding LpxR, compared with the bulk lipid region. **Figure S3c** acts as a graphical legend for **S3a** and **S3b**. This greater level of disorder supports our observation that the membrane is thinner in the 1.2 nm surrounding the

protein; LpxR disrupts the membrane, causing localised thinning and disorder of lipid acyl tails.

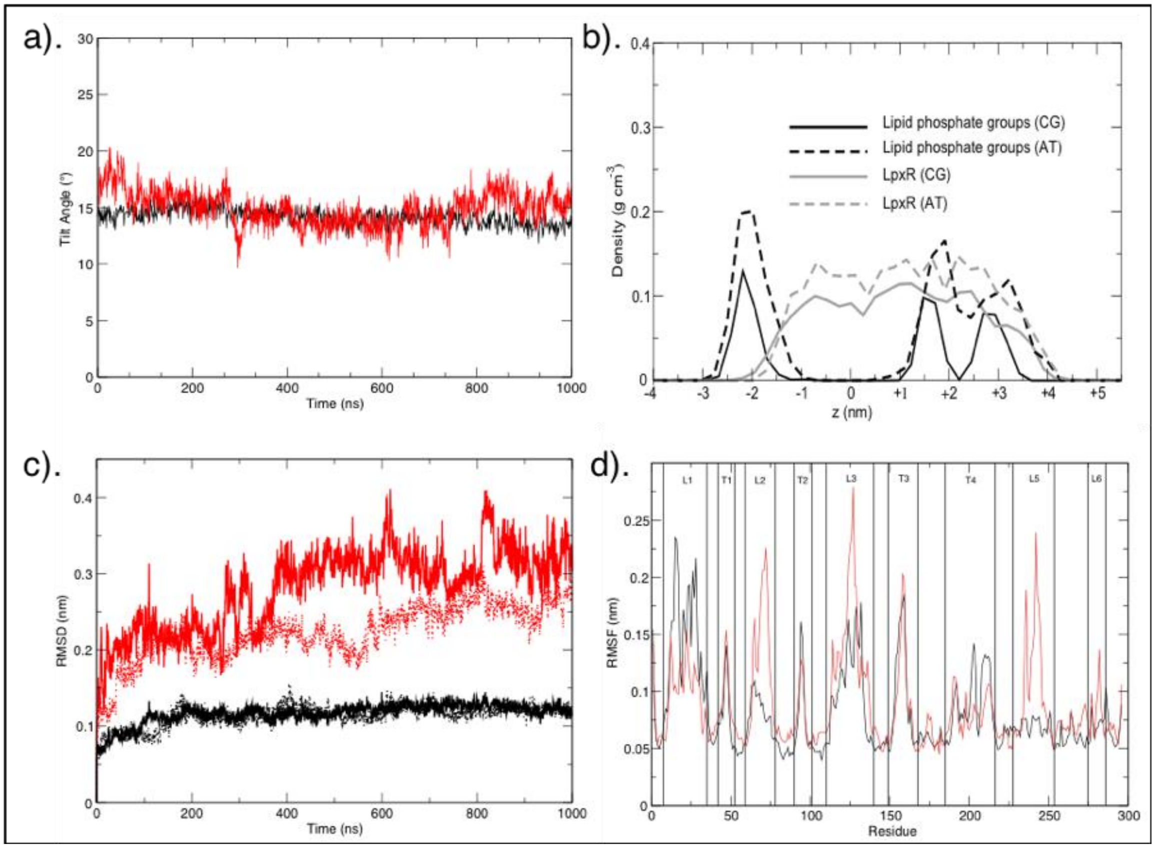

Figure S4. a). Protein barrel tilt angle relative to bilayer normal over 1 μs of coarse-grain (black) or united-atom (red) simulation **b).** Relative densities of protein and lipid headgroups, coarse-grain (CG) and atomistic (AT), along the z axis **c).** RMSD of protein beta barrel (black) and alpha helices (red) in apo (solid line) and ligand-bound (broken line) systems **d).** RMSF of protein in apo (black) and ligand-bound (red) systems.

To ensure the orientation of protein in AT systems corresponded with their CG counterparts, both the tilt angle of the barrel principal axis and system partial densities were analysed and compared. The tilt angle of the principal axis of the

beta barrel was derived from the centre of mass of residues at the top and bottom of the barrel, relative to the bilayer normal.

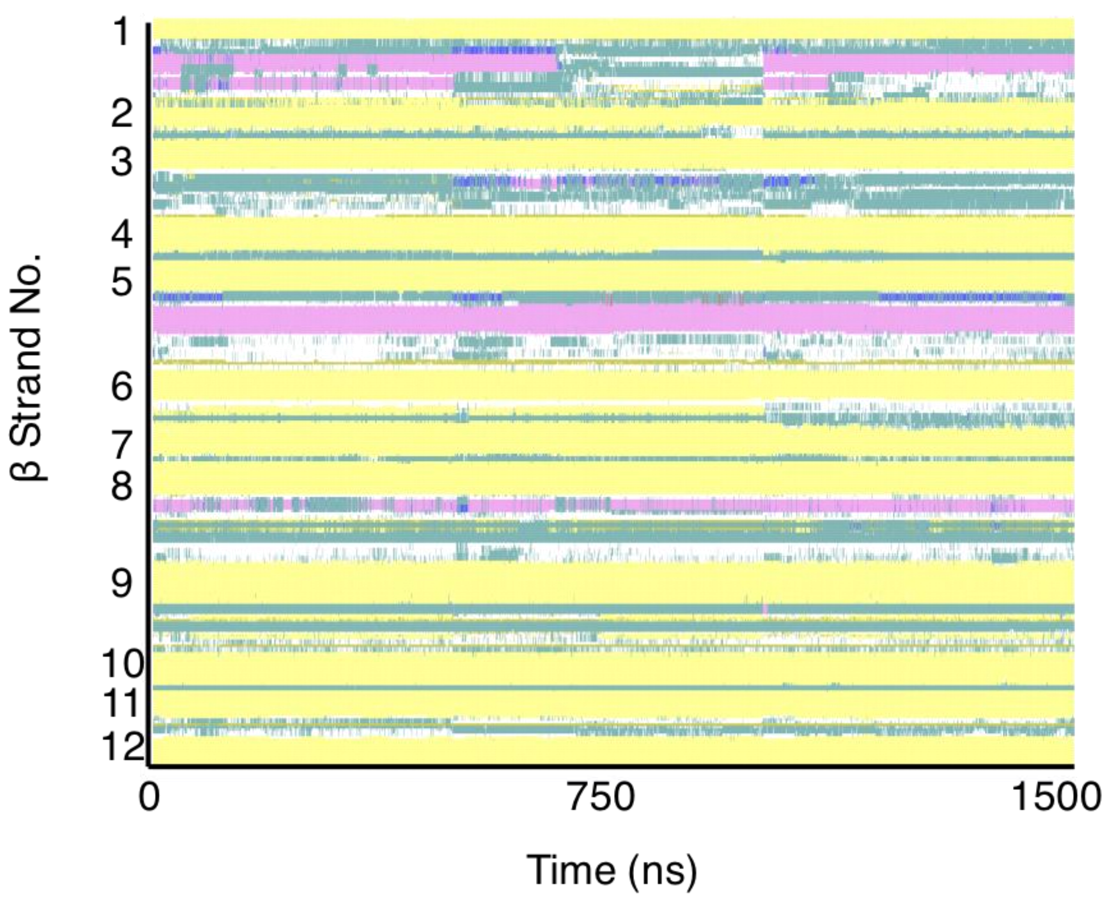

Figure S5. Depicting the secondary structure of residues of LpxR across 1500 ns of simulation. β strand numbers labelled for clarity. Colouring as follows; yellow β sheet, pink α helix, green turn, blue 3_{10} helix, white coil, red π helix and brown isolated bridge. Secondary structure analysis was performed using the VMD script on the full length concatenated 1500 ns of protein from micelle simulations.

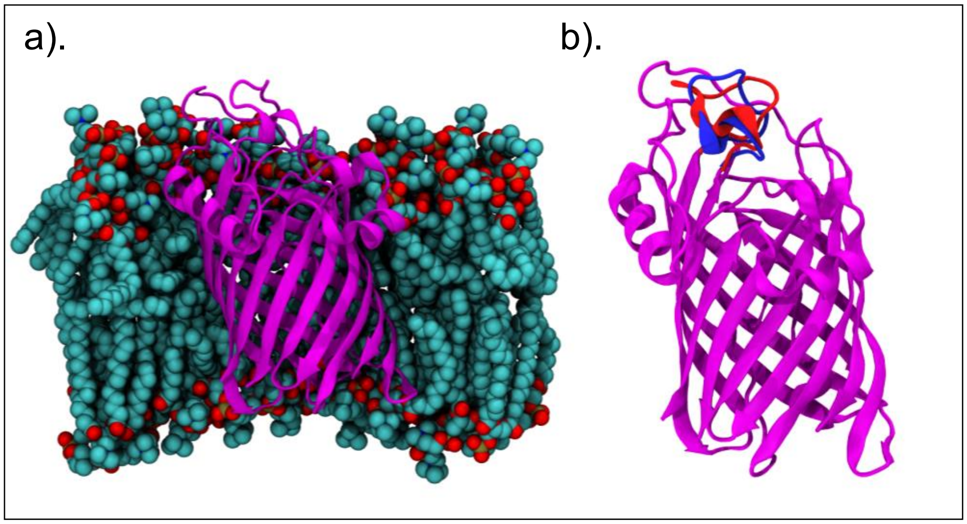

Figure S6. a). A representative snapshot of the closed conformation of LpxR in DPPC bilayer, with water and some lipids are removed for clarity **b).** An overlay of extracellular loop L2 throughout DPPC bilayer simulations. Blue denotes position of L2 at 0 μs and red at 1 μs.

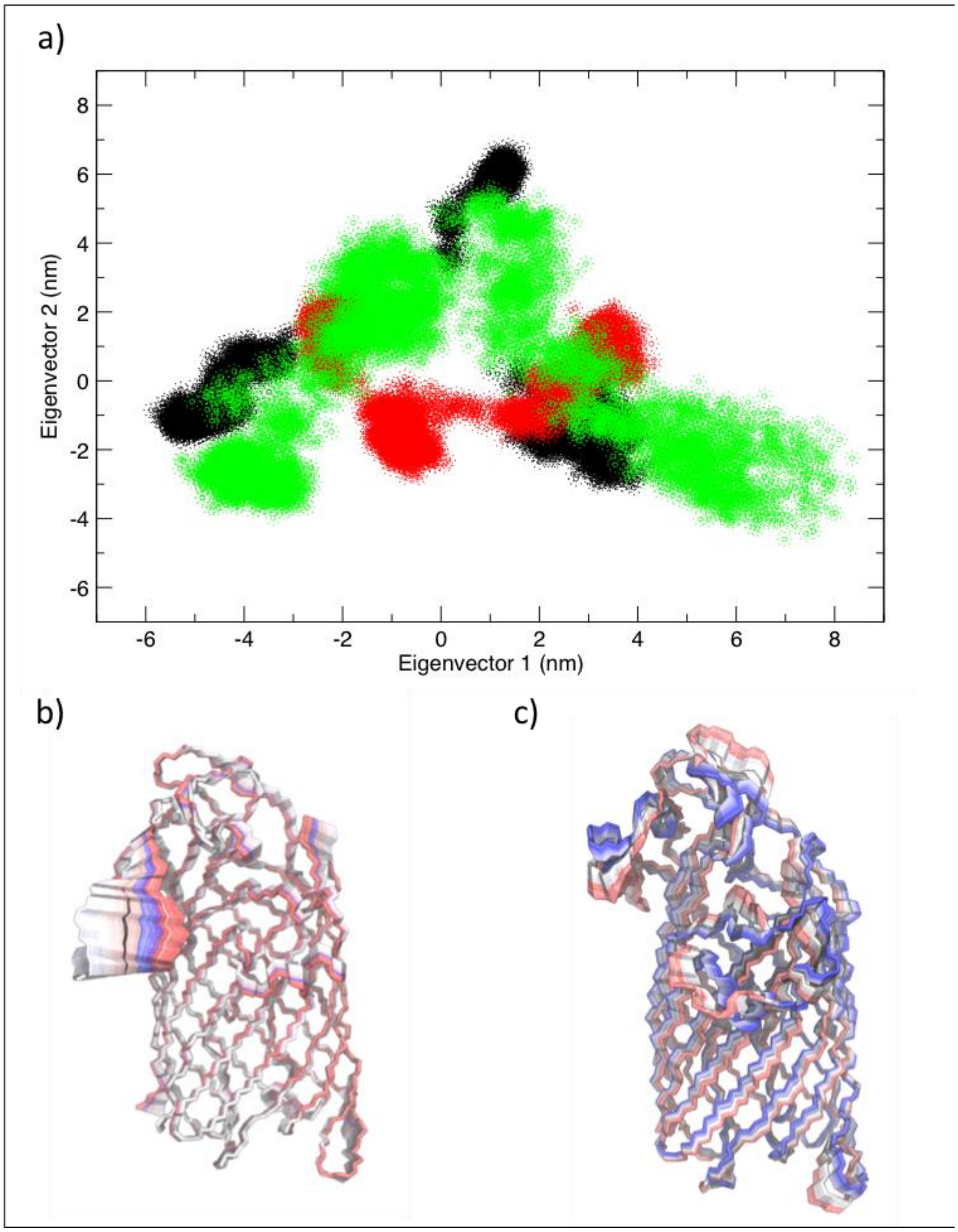

Figure S7. a). A 2d projection of the first two principal components of protein backbone movement. Black points denote LpxR in asymmetric Ra LPS membrane, red in DPPC membrane and green in DPC micelle **b).** The motion of the protein backbone between two extreme projections described by the first principal component with protein conformations overlaid every 20 ps. Protein backbone trajectory taken from simulations with protein in DPPC membrane. The image is coloured on a BWR scale (blue at the start of the simulation, through white, to red at the end of the simulation) **c).** Identical to **b)**, but protein backbone

trajectory taken from simulations with protein in Ra LPS membrane.

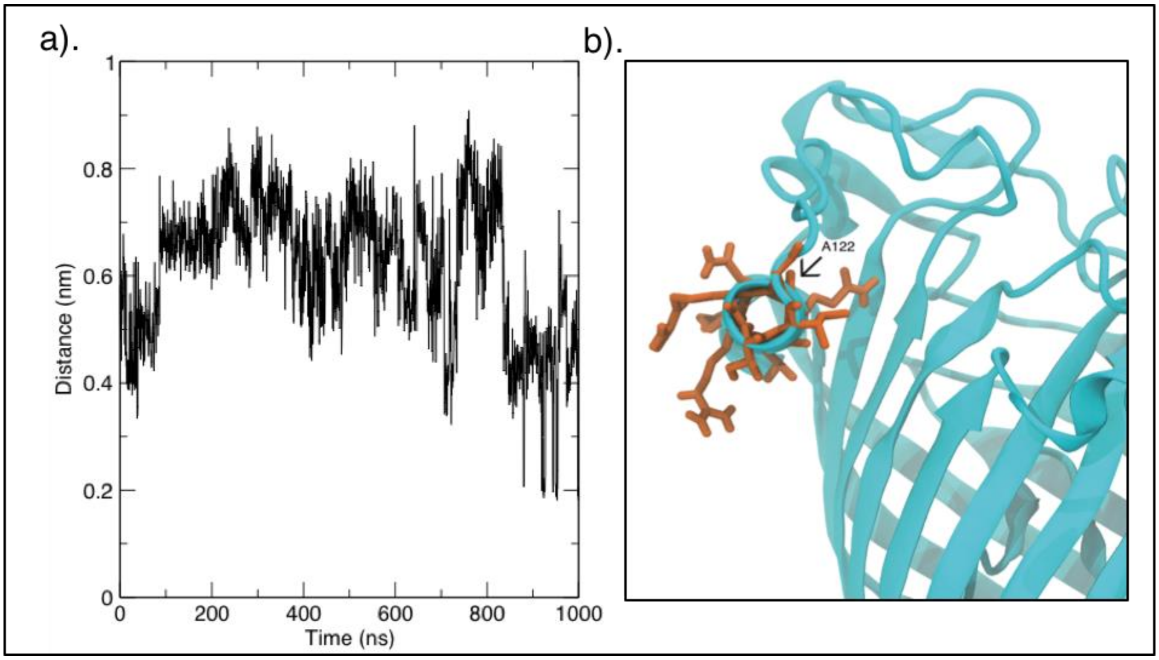

Figure S8. a). Distance between H122 and E128 centre of mass to indicate possibility of hydrogen bond formation between the two residues. **b).** Helix of extracellular loop L3 with H122A mutation after 1 μs. Protein backbone coloured in cyan, residues 110-126 in orange.

As mentioned in the main text, we saw did not see evidence of a stable hydrogen bond between H122 and E128 during or simulations. We show in **Figure S8a** the distance between the centre of mass of H122 and E128 during the "model" simulation at 323 K, and how the two residues do not spend large periods of the simulation close enough to form a hydrogen bond.

Also in the main text is the claim that the H122A mutation does not affect the conformational stability of the alpha helix of extracellular loop L3. This is demonstrated in **Figure S8b**.

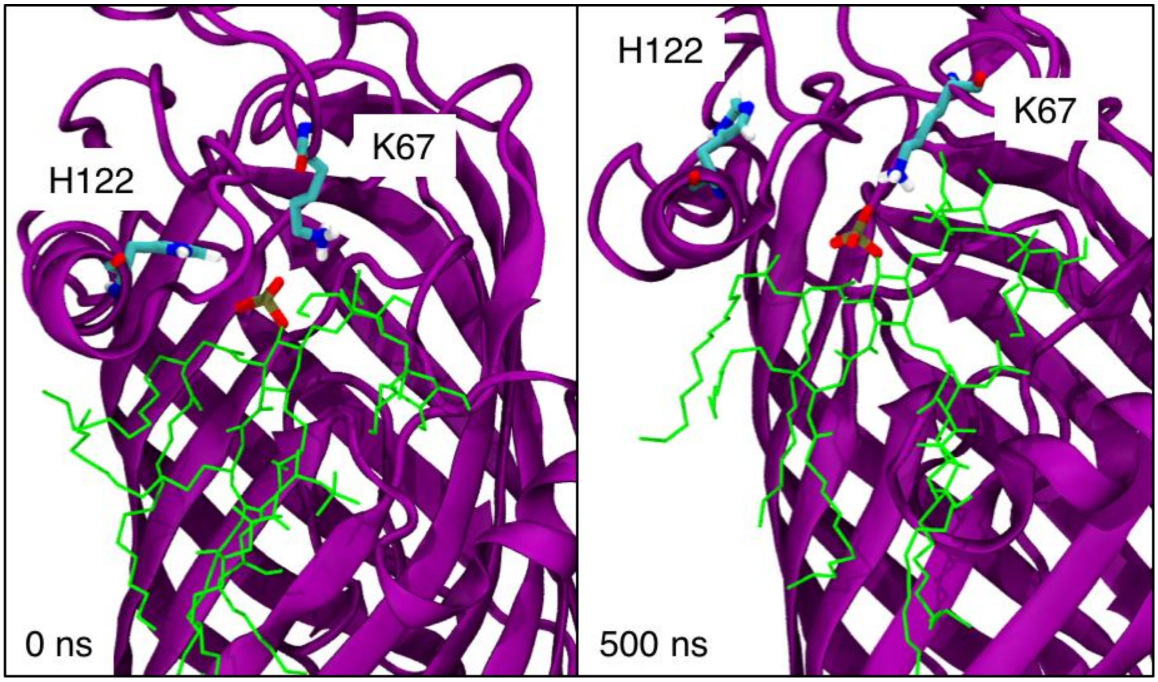

Figure S9. Movement of H122 away from the scissile bond accompanied by ‘snorkelling’ of K67 towards the 1’ phosphate of Re LPS bound to LpxR. Protein in purple and components of Re LPS other than the 1’ phosphate in lime green. The two images represent time points of 0 ns and 500 ns. Image generated from representative model ligand-bound system at 323 K.

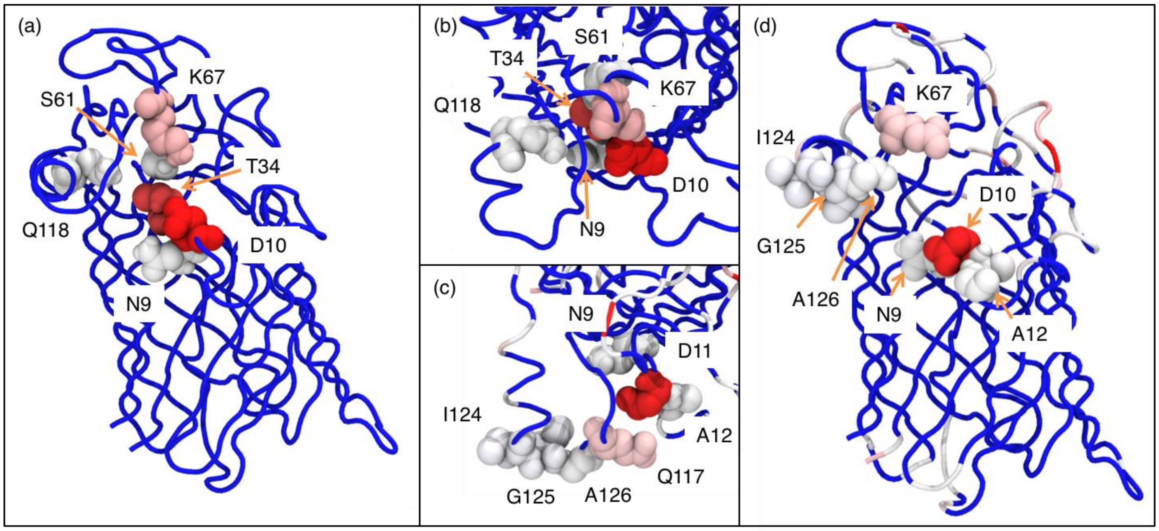

Figure S10. Heat-map of protein residue contacts with cations in the LPS binding site, with BWR colour scale. Contact defined as an interatomic distance of $\leq$ 0.4 nm **a).** Residues in contact with Ca^{2+} ions over 1μs simulation at 323 K. **b)** same as **a)** but from a top-down viewpoint. **c)** Residues in contact with Mg^{2+} ions over 1μs simulation at 323 K, with a top-down view of the binding site. **d)** same as **c)** but viewing the binding site of the protein from the side.

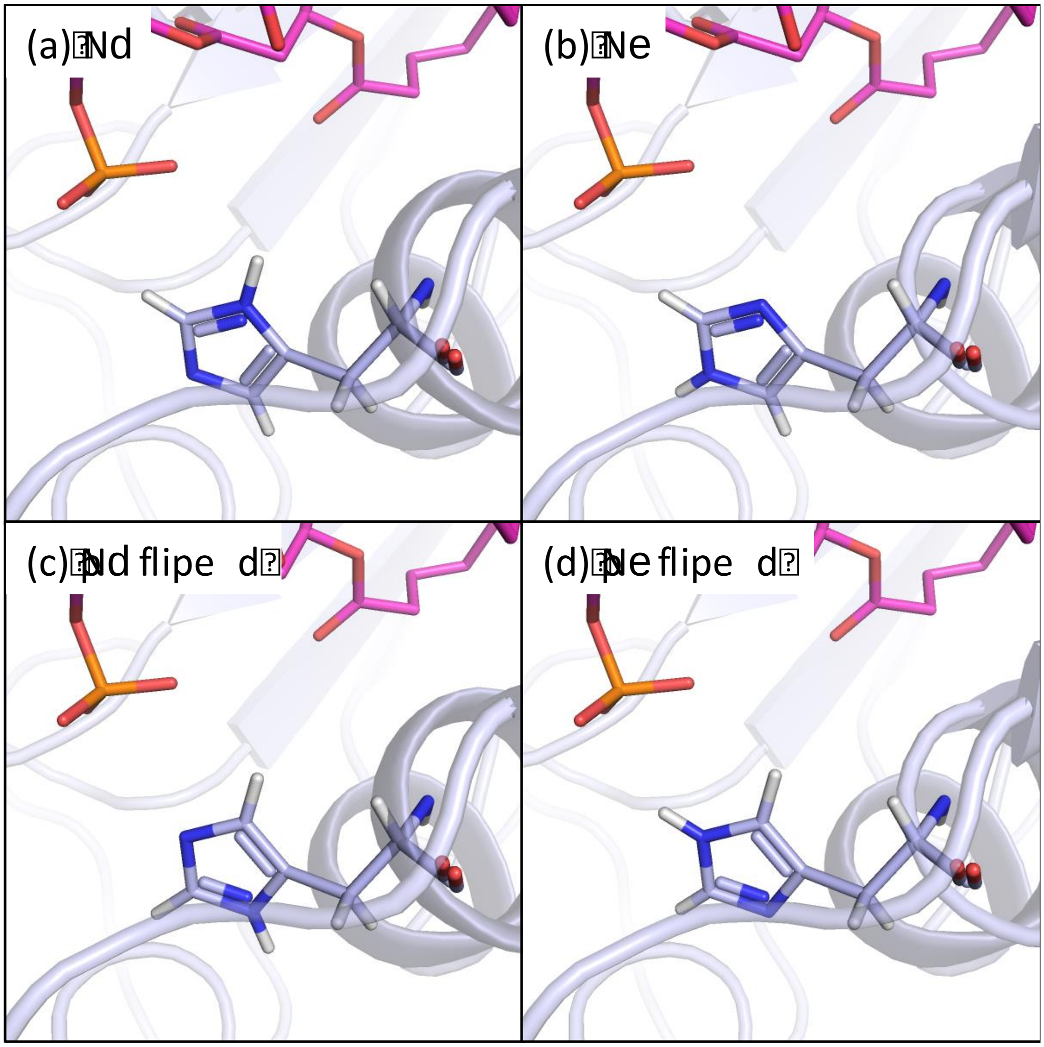

Figure S11. All starting orientation of H122 for GCMC titration simulations; **a).** Nδ **b).** Nε **c).** Nδ flipped and **d).** Nε protonated. Protein is shown in light blue, with histidine and REMP shown in stick representation, coloured light blue and pink

respectively.

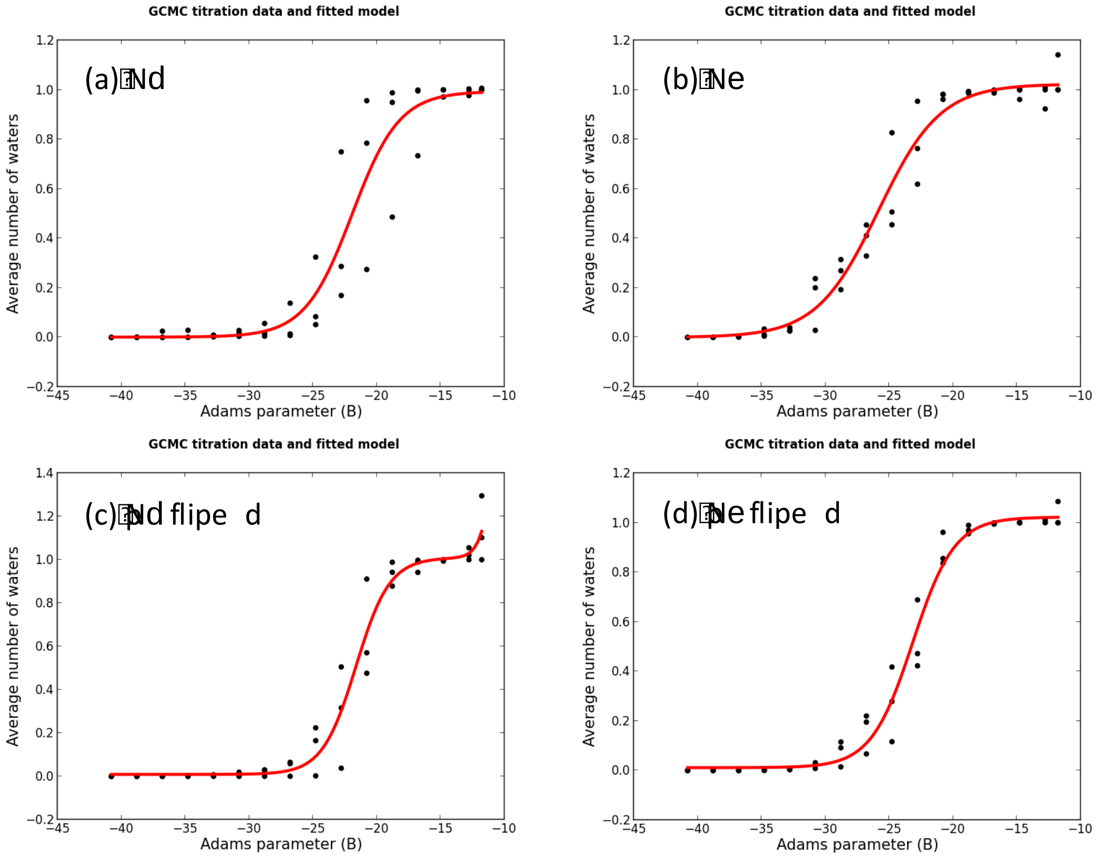

Figure S12. GCMC titration curves for the binding free energy calculations for water molecules with **a)**. Nδ **b)**. Nε **c)**. Nδ flipped and **d)**. Nε protonated H122 protein.

Titration curves show the average water occupancy for a simulation at each B value. The higher the B value, the higher the average water occupancy. A logistic function has been fit to the data (shown in red), from which the integral of this can be used to calculate the binding free energy of the number of water

molecules (a single water molecule in this case). This can be calculated using the GCI equation. The GCI equation relates the above results and their titration curves to the binding free energies presented in the manuscript.

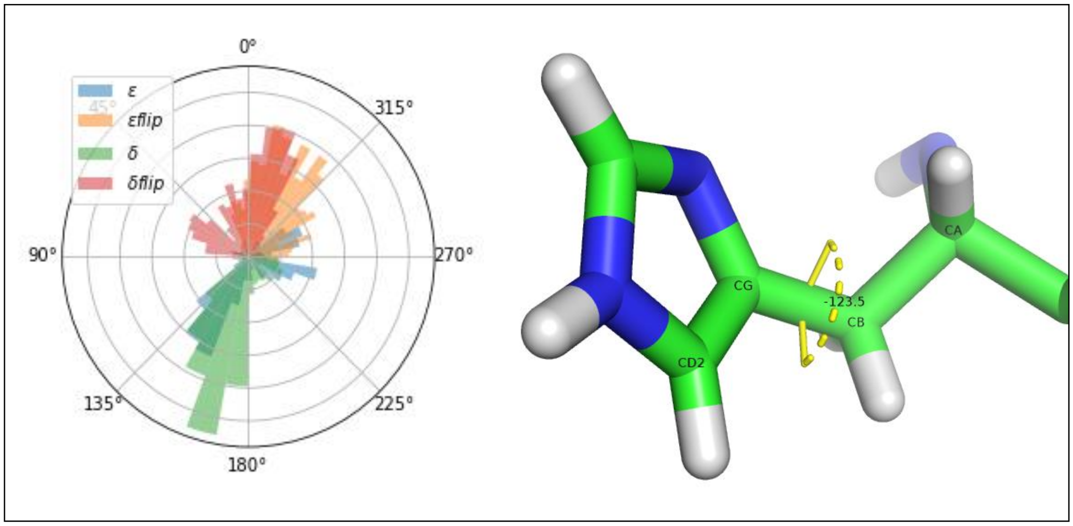

Figure S13. Sampling of H122 rotamers observed in GCMC simulations with dihedral angle illustrated. Sampling of epsilon and delta is mostly observed between 135-180°, while the flipped simulations are observed to sample mostly between 315-360°.
